# Modulation Depth Discrimination by Cochlear Implant Users

**DOI:** 10.1007/s10162-022-00834-6

**Published:** 2022-01-26

**Authors:** Jessica J. M. Monaghan, Robert P. Carlyon, John M. Deeks

**Affiliations:** 1 Macquarie University, The Australian Hearing Hub, NSW 2109 Sydney, Australia; 2 National Acoustic Laboratories, The Australian Hearing Hub, Sydney, NSW 2109 Australia; 3 Cambridge Hearing Group, Medical Research Council Cognition and Brain Sciences Unit, University of Cambridge, 15 Chaucer Road, Cambridge, CB2 7EF UK

**Keywords:** Cochlear implant (CI), Modulation depth discrimination, Amplitude modulation sensitivity, Masking, Temporal processing, Temporal window, Envelope

## Abstract

Cochlear implants (CIs) convey the amplitude envelope of speech by modulating high-rate pulse trains. However, not all of the envelope may be necessary to perceive amplitude modulations (AMs); the effective envelope depth may be limited by forward and backward masking from the envelope peaks. Three experiments used modulated pulse trains to measure which portions of the envelope can be effectively processed by CI users as a function of AM frequency. Experiment 1 used a three-interval forced-choice task to test the ability of CI users to discriminate less-modulated pulse trains from a fully modulated standard, without controlling for loudness. The stimuli in experiment 2 were identical, but a two-interval task was used in which participants were required to choose the less-modulated interval, ignoring loudness. Catch trials, in which judgements based on level or modulation depth would give opposing answers, were included. Experiment 3 employed novel stimuli whose modulation envelope could be modified below a variable point in the dynamic range, without changing the loudness of the stimulus. Overall, results showed that substantial portions of the envelope are not accurately encoded by CI users. In experiment 1, where loudness cues were available, participants on average were insensitive to changes in the bottom 30% of their dynamic range. In experiment 2, where loudness was controlled, participants appeared insensitive to changes in the bottom 50% of the dynamic range. In experiment 3, participants were insensitive to changes in the bottom 80% of the dynamic range. We discuss potential reasons for this insensitivity and implications for CI speech-processing strategies.

## INTRODUCTION

In most contemporary processing strategies, cochlear implants (CIs) convey information about the sound-energy envelope in each frequency channel by modulating the amplitudes of high-rate pulse trains. The ability of CI users to process such modulations has been widely studied using the modulation detection threshold (MDT) — the smallest depth of modulation that can be discriminated from a train of unmodulated electrical pulses, applied to a single CI electrode. MDTs can reveal basic aspects of the auditory processing of electrical stimulation. For example, variation in the MDT as a function of modulation rate defines the temporal modulation transfer function, which is a measure commonly used to describe the limitations on temporal processing by CI users. Performance on this task is often very good, and, at least at high levels and for slow modulations, often corresponds to only 1–2% of the subject’s dynamic range, defined on a decibel scale (Chatterjee and Oberzut 2011; Fraser and McKay 2012; Green et al. 2012; Shannon 1992). These small MDTs suggest that CI listeners can accurately encode amplitude modulations near the peaks of the amplitude envelope.

In contrast to the well-established literature investigating MDTs, relatively little is known about how accurately CI listeners process modulations near the troughs of the envelope. In normal hearing listeners, information in the envelope troughs has been shown to have a significant contribution to speech intelligibility. Drullman (1995a) demonstrated that removing the speech modulations from the troughs of speech in noise resulted in a 2-dB increase in the speech reception threshold (SRT). Furthermore, Drullman (1995b) found that 75% speech intelligibility was retained for sentences where peaks above the median envelope level were removed, leaving speech information only in the troughs. More recently, Stone et al. (2010) found that intensities of between + 10 and − 20 dB relative to the channel RMS were most important for intelligibility, with the most important levels being close to − 5 dB. Clearly, the ability of CI listeners to understand speech will depend on the audibility and discriminability of this information. To investigate the sensitivity of CI users to information in envelope troughs, the present study measures their ability to discriminate fully modulated from less-modulated pulse trains applied to one channel of a CI. We believe that studying modulation processing at supra-threshold depths can not only shed light on the processing of modulation depths in a way that is more relevant to the perception of everyday sounds, but can also inform the development and evaluation of novel processing strategies. Data obtained with a number of such strategies suggest potential benefits from sharpening the modulation envelope (Green et al. 2004; Laneau et al. 2006; Monaghan and Seeber 2016; Vandali et al. 2005; Vandali and van Hoesel 2011), replacing it entirely with single pulses at each envelope maximum (Smith et al. 2014), or deleting pulses that would likely be masked by adjacent higher-amplitude pulses (Kludt et al. 2021; Lamping et al. 2020). These studies have improved, for example, the perception of modulation rate, the perception of speech in noise, and, in bilaterally implanted listeners, of interaural time differences (ITDs). However, they necessarily remove information about modulation depth and more subtle differences in modulation shape that may be important overall for good listening performance. Our experiments represent a first step in quantifying how much of the dynamic range (DR) contributes to sensitivity to amplitude modulation in CI listeners, and address whether the potential benefits of these sparser CI processing methods can be preserved whilst simultaneously maintaining the perception of differences in modulation depth. We also compare our results with those of the only other studies of CI modulation depth discrimination of which we are aware (Busby et al. 1993; Gomersall et al. 2016).

Three different experimental paradigms were employed using a high-rate pulse train modulated at 100% of its DR, delivered on a single electrode at a wide range of modulation frequencies, ranging from the 3–8-Hz speech syllable rate (Raphael 2008) to the 15–30-Hz rate corresponding to phonemes (Liberman et al. 1967), to faster (62.5–250 Hz) modulations that convey pitch (Rosen et al. 1992). First, an odd-one-out procedure was employed in which the listener could exploit any cue to detect the signal interval. The signal stimulus had a smaller modulation depth than the standard stimulus, and this depth of modulation was varied to find a threshold using an adaptive tracking procedure. As with acoustic stimuli, electrical pulses are subject to masking from preceding stimuli — forward masking (Lüscher and Zwislocki 1947; Plomp 1964; Shannon 1990) — and from trailing stimuli — backward masking (Blamey and Dooley 1993; Oxenham and Moore 1994). We therefore expected the modulation minima to be undetectable, and for the proportion of the stimulus that is undetectable to increase with increasing modulation rate; this did indeed occur. Experiment 2 also measured modulation depth discrimination, but employed a paradigm whereby the use of potential loudness cues could be minimised and closely monitored. To do so, we used a two-interval forced-choice task, instructing listeners to ignore loudness, and checked whether they had indeed done so by including ‘catch’ trials in which the less-modulated signal was presented at an overall lower level than the 100% modulated standard. In a third paradigm, loudness cues were eliminated by using a stimulus where, rather than varying modulation depth by changing the level of the minima of a sinusoidal envelope, the shape of the envelope below a criterion level was changed, and the level of this criterion was adaptively varied. This experiment therefore provided information on the proportion of the dynamic range over which listeners can discriminate changes in the shape of the amplitude envelope.

## EXPERIMENT 1: MODULATION DEPTH DISCRIMINATION AS A FUNCTION OF AMPLITUDE MODULATION RATE

### Method

#### Participants

Ten adult, post-lingually deafened users of devices manufactured by Cochlear Ltd took part. Five (C1, C2, C4, C5, C6) were recruited at the MRC Cognition and Brain Sciences Unit in Cambridge, UK, and five (M1, M2, M3, M4, M6) at Macquarie University in Sydney, Australia. Participant information is displayed in Table 1. The table includes details of an additional listener, C7, who took part in experiment 3. The experiments were approved by the ethics boards of the Faculty of Human Sciences at Macquarie University and by the Local Research Ethics Committee for the East of England.

**Table 1:** Participant information

Participant	Age	Duration of deafness	Years of implant use	Thresh CUs	MCL CUs	DR
C1	69	48	14	108	174	66
C2	75	10	15	96	147	51
C4	78	5	3.5	138	170	32
C5	67	1.5	3.5	102	145	43
C6	75	15	14	135	172	37
C7	70	2	4	114	165	51
M1	62	28	5	84	147	63
M2	71	2	18	144	183	39
M3	62	38	4	90	167	77
M4	76	51	3	102	198	96
M6	64	8	7	65	162	97

#### Stimuli and Hardware

**Fig. 1:**
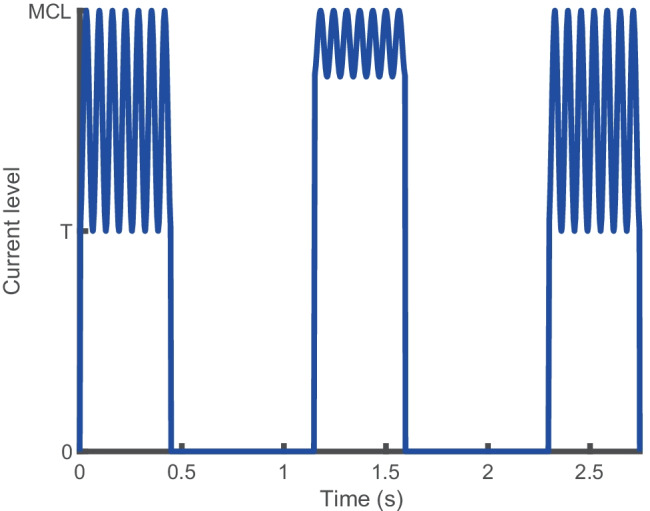
Modulation envelopes for the three stimulus intervals in exp. 1, for a trial where the signal is in interval 2, and with a modulation rate of 15.625 Hz

All stimuli were generated in Matlab and delivered via a laboratory-owned Freedom (SP12) processor and Nucleus Implant Communicator 2 (NIC2) research software routines provided by the manufacturer. Stimuli were trains of anodic-leading symmetric biphasic pulses presented in monopolar mode (MP1 + 2) to electrode 16 (mapped to 938–1063 Hz). The phase duration was 43 µs, and there was an 8-μs inter-phase gap. The pulse rate was 1000 pulses per second (pps), and the overall duration of the pulse trains was 0.448 s. Unmodulated pulse trains were used to estimate the threshold and ‘most comfortable loudness’ levels (MCLs), as described below. For the modulated stimuli used in the main experiments, the stimuli were sinusoidally amplitude modulated at a rate of 15.625, 31.25, 62.5, 125, or 250 Hz. A restriction in the buffer size of the SP12 processor limited the lowest modulation frequency that could be tested whilst still allowing for at least 7 modulation cycles. The overall duration of 0.448 s gave an integer number of modulation cycles of 7, 14, 28, 56, 112, and 224 at each rate, respectively. The starting phase of modulation was always zero. Amplitude modulation for all rates was defined in terms of current units (CUs), with peak amplitude corresponding to MCL for an unmodulated pulse train for that participant. A change at a level of 1 CU corresponds to an approximately 0.15-dB difference in current. A modulation depth for a given participant of 100% consisted of a stimulus whose envelope amplitude varied between the threshold (T-level) and MCL, as illustrated by the first and third stimuli in Fig. 1. The envelope for a signal with a modulation depth of 30% varied from MCL to 30% of that range as illustrated by the middle stimulus in Fig. 1. No adjustment was made for average current, so that a 100% modulated pulse train would likely have reduced loudness relative to a less-modulated pulse train, since the RMS current level was lower for the fully modulated condition, but the peak level was fixed. McKay et al. (2003) and McKay and Henshall (2010) found that for CI users, loudness was dependent on both peak and RMS current level, with peak level becoming the dominant influence at higher absolute current levels. All stimuli were checked using a test implant and a digital storage oscilloscope. Contact impedances were checked at the start and end of every session.

#### Procedure

Thresholds and MCLs were determined for each subject using loudness estimation and employed unmodulated pulse trains with the same parameters as the stimuli used in the main part of the experiment. Subjects were asked to estimate the loudness of pulse trains on a chart, indicating loudness on a scale from 0 (‘off’) to 10 (‘too loud’). The level of the pulse train was increased until the participant indicated a rating of 1 (‘just noticeable’), which was recorded as T-level. The level was further increased until loudness level 6 (‘most comfortable’), and then 7 (‘loud but comfortable’) was reported. The midpoint of the range of current levels indicated as ‘6’ was recorded as MCL. The procedure was repeated two or three times depending on the consistency of the values, and the results were averaged to give the Ts and MCLs used in the experiments. The DR was defined as the range of current levels between T and MCL.

Modulation depth discrimination was measured using a 3-interval 2-alternative forced-choice paradigm with a 3-up, 1-down rule. The standard stimulus was 100% modulated (defined according to the subject’s DR), and appeared in interval 1, and also in either interval 2 or 3. Subjects were instructed to indicate which of sound 2 or 3 was the odd one out, and that they could use any cue to guide their answers. Correct-answer feedback was provided after each trial. The signal stimulus occurred in either interval 2 or 3, and was initially unmodulated or had very little modulation. Signal modulation depth (with peak level fixed at MCL) was increased after every two consecutive correct answers and decreased after every incorrect answer, with the change from increasing to decreasing modulation or vice versa defined as a turn-point. The modulation depth step size was 3 CUs for the first two turn-points, and 1 CU for the last four turn-points. For each adaptive run, a total of six turn-points was measured, with the last four turn-points averaged to represent the run. Between 2 and 4 adaptive runs were made for each AM rate, and the average was taken to represent each condition. The inter-stimulus interval was 0.7 s. Each trial started 1 s after the subject’s response to the previous trial. Modulation depth discrimination was measured for rates 15.625, 31.25, 62.5, 125, and 250 Hz. Thresholds for the different modulation frequencies were recorded in blocks of ≥ 62.5 Hz (pitch-like cues) and < 62.5 Hz (wobble cues) so as to minimise the number of times that participants had to switch between different cue types. The order of modulation frequencies within blocks and the order of blocks were randomised.

### Results

**Fig. 2:**
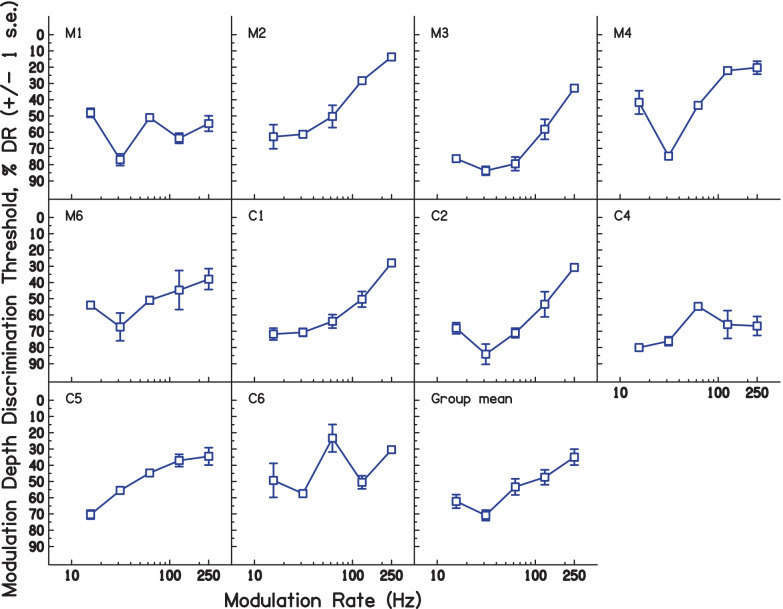
Modulation depth thresholds expressed in terms of % DR, for each subject in experiment 1. The bottom-right panel shows the mean of all subjects. Individual subject data show ± 1 s.e. of the mean of trials making up each condition. Group mean data show ± 1 s.e. of the group mean

Figure 2 shows the modulation depth discrimination thresholds (MDDTs), expressed as a percentage of dynamic range for each subject, with the average across subjects shown in the bottom right-hand panel. Note that discrimination thresholds were measured as the difference from 100% modulation; i.e. a reduction in AM depth is measured. Thus, thresholds with larger numbers indicate better performance (smaller just noticeable difference). For example, C1’s MDDT of about 70% at 31.25 Hz means a 30% reduction (from 100%) was required to discriminate a fully modulated stimulus from a less-modulated one; the MDDT of about 27% at 250 Hz meant that a 73% reduction was required.

In general, detection thresholds worsened with increasing modulation rate, consistent with the expected influence of forward (and, to a lesser extent, backward) masking and broadly consistent with the performance of normal hearing listeners in AM detection tasks (Ewert and Dau 2004; Kohlrausch et al. 2000; Viemeister 1979). Performance was also relatively poor at the lowest modulation rate examined, 15.625 Hz, possibly because there were only 7 modulation cycles present for this modulation rate. There is some evidence that a minimum number of modulation cycles is required for accurate AM depth discrimination (Gomersall et al. 2016; Lee and Bacon 1997).

It is apparent that the pattern of results shown in Fig. 2 varies somewhat across subjects. One source of variation is that the above-mentioned increase in MDDT as the modulation rate drops from 31.25 to 15.625 Hz occurs only for some participants, being most marked for M1, M4, and C2, and reduced or absent for other listeners. Another feature is that three subjects — M1, C4, and C6 — show higher MDDTs at a rate of 61.25 Hz than at either of the two immediately adjacent rates. Despite these differences, the main effect of modulation rate, assessed by fitting a linear mixed model (LMM) with modulation rate as a continuous variable and a random intercept for each subject, was highly significant (*χ*^2^(1) = 17.04, *p* < 0.001, likelihood-ratio test).

## EXPERIMENT 2: MODULATION DEPTH DISCRIMINATION AS A FUNCTION OF AMPLITUDE MODULATION RATE WITH ADDED CHECKS FOR USE OF LOUDNESS CUES

### Rationale

Experiment 2 measured MDDTs whilst controlling for and monitoring the potential use of loudness cues. In modulation *detection* tasks, it is possible to control for loudness cues by roving the level of the unmodulated stimulus (Fraser and McKay 2012; Galvin et al. 2014). Unfortunately, this is not appropriate for the AM depth discrimination task, in which both stimuli in each trial are modulated; this is because roving the overall level may also rove the perceived modulation depth, which is the percept that we are trying to measure. For example, for the 100% modulated standard stimulus, reducing the overall level will necessarily reduce the difference in amplitude between the peak of the modulator and the listener’s detection threshold for an unmodulated pulse train, potentially reducing the perceived modulation depth (although the modulation depth as typically defined would still technically be 100%). This will also happen for signal stimuli with large modulation depths. Instead, we minimised the use of loudness cues by using 2-interval trials and instructing subjects to ignore loudness, and checked whether they followed those instructions by inserting catch trials.

### Method

#### Participants

The same participants as in exp. 1, except for C5, took part.

#### Stimuli

The same stimuli as those for exp. 1 were used, with the exception of ‘catch trial’ stimuli that were presented on about 15% of occasions. These are described below.

#### Procedure

Prior to the start of exp. 2, we checked that the thresholds and MCLs obtained for exp. 1 were still valid for each listener, using the same loudness estimation procedure. This was true for each listener, and so the same levels were used for exp. 2.

Modulation depth discrimination was measured using a 2-interval 2-alternative forced-choice task and an adaptive procedure that employed a 3-up, 1-down rule. The standard stimulus was 100% modulated (according to each subject’s DR). The signal stimulus was initially unmodulated or had very little modulation. Signal modulation depth (with peak level fixed at MCL) was adaptively increased with successive correct answers to find threshold. For AM rates of 15.625 and 31.25 Hz, participants were instructed to indicate which of sound 1 or 2 was ‘the more wobbly’. For AM rates of 62.5, 125, and 250 Hz, participants were instructed to indicate which interval had the higher pitch; this instruction was based on the finding that the pitch of high-rate pulse trains typically increases with decreasing modulation depth (Vandali et al. 2013). Participants were instructed that they should ignore any differences in loudness and concentrate on ‘wobble’ or pitch only. Correct-answer feedback was provided for 85% of trials. A total of 8 turn-points were measured, with the last 6 averaged. As in experiment 1, the modulation depth step size was 3 CUs for the first two turn-points, and 1 CU for the last four turn-points, and the inter-stimulus interval was 0.7 s.

**Fig. 3:**
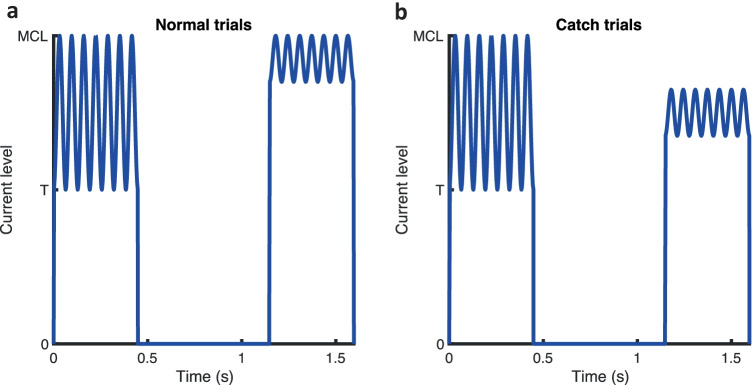
Stimulus envelopes for experiment 2 for **(a)** regular trials and **(b)** catch trials

For each procedure, after 2 turn-points were measured, a number of 2-interval catch trials were presented in addition to the adaptive procedure trials. For these catch trials (and 15% of conventional trials), feedback was not given, and participant responses did not contribute to the course of the adaptive procedure. Catch trials occurred immediately after reversals in the adaptive track and randomly in 15% of other trials, resulting in 9–11 catch trials per adaptive run. In catch trials the standard was modulated by 100% and the signal had a modulation depth corresponding to that currently set in the adaptive procedure, but with the modulation applied at a level of 50% of the participant’s DR. The stimulus envelope is illustrated in Fig. 3 for a signal with modulation depth of 30%. In contrast to the conventional trials (see Fig. 3a), in which the less-modulated signal was perceived as being louder than the standard, in catch trials (see Fig. 3b), the signal was perceived as being quieter than the standard, due to its lower RMS and peak amplitude (McKay and Henshall 2010). For the catch trials, it was assumed that if a participant did not choose the correct answer significantly more frequently than would be predicted by chance (50%), it was because they were at least somewhat basing their answers on loudness cues (by always choosing the quieter stimulus rather than the more modulated one). Participants were instructed that occasionally they would hear a ‘soft’ trial, and that, as for all other trials, they should ignore any differences in loudness and concentrate on wobble or pitch only, depending on the modulation rate. Informal reports from subjects indicated that catch trials were perceived as soft compared to main trials, but still easily audible.

### Results

**Fig. 4:**
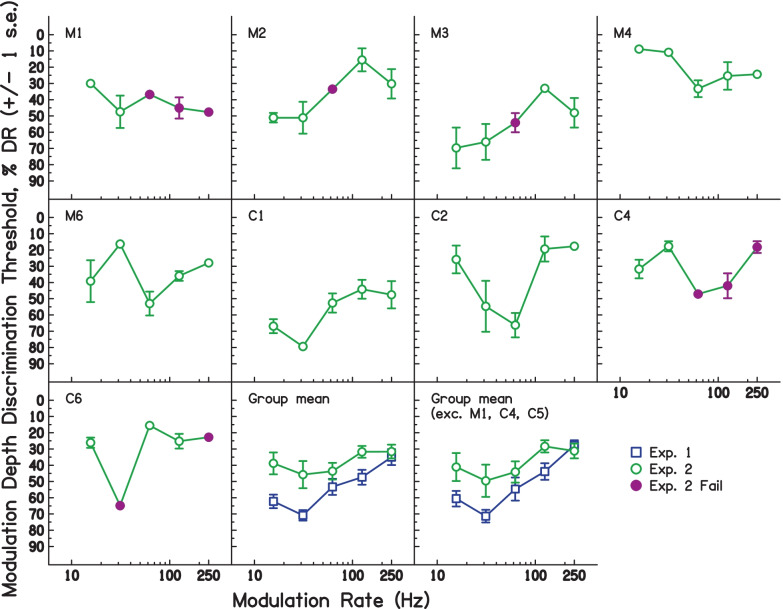
Modulation depth thresholds expressed in terms of % DR, for each subject in experiment 2. The bottom-right panel shows the mean of all subjects. Individual subject data show ± 1 s.e. of the mean of trials making up each condition. Group mean data show ± 1 s.e. of the group mean

The results of experiment 2 are shown in Fig. 4. Data for individual subjects are shown in each panel, with open circles denoting measures where the number of ‘correct’ catch trials was significantly greater than 50% (*p* < 0.05 or greater; binomial distribution test); we consider these thresholds to be primarily based largely on wobble or pitch cues, rather than on overall loudness cues. Filled circles indicate when the proportion of correct catch trials was not significantly higher than 50%, and these thresholds may have been primarily affected by overall loudness cues. The bottom-centre panel shows the mean across all subjects for experiment 1 and experiment 2. The bottom-right panel shows the mean across those subjects where the thresholds were valid (proportion of catch trials significantly > 50%) for over half of the modulation frequencies (i.e. excluding M1 and C4); this panel also excluded C5, who did not take part in experiment 2.

The data indicate that, in 78% of subject/modulation-rate combinations, loudness was largely ignored. However, the catch-trial scores for M1 and C4 in the higher-rate conditions were not significantly above chance level, indicating that they may have been at least partially using level cues in their judgements in the majority of conditions. Their data are accordingly excluded from the grand means, as noted above. As in exp. 1, the pattern of results differed somewhat across listeners, but a significant main effect of modulation rate was again observed, here using a LMM excluding any runs where the number of failed catch trials indicated that participants were influenced by loudness (*χ*^2^(1) = 6.33, *p* = 0.0118). A LMM including experiment as a factor and modulation rate as a continuous variable was fitted to compare thresholds from exp. 1 and exp. 2, and revealed a significant effect of experiment (*χ*^2^(1) = 41.82, *p* < 0.001), indicating thresholds were significantly worse in exp. 2. There was a significant overall effect of modulation rate (*χ*^2^(1) = 36.12, *p* < 0.001), and the interaction between modulation rate and experiment was also significant (*χ*^2^(1) = 15.59, *p* < 0.001).

The poorer performance in exp. 2 suggests that participants might have been exploiting differences in sound level to make their judgements in exp. 1; removing or reducing access to this cue reduced overall discrimination performance. However, this explanation would predict that the difference in MDDTs between the two experiments should be largest at high modulation rates, where the MDDTs — and hence also differences in loudness — should be greater. However, inspection of the mean data (bottom-right panel of Fig. 4) does not support this hypothesis; indeed, the difference appears slightly greater at the lower modulation frequencies. An alternative explanation is that the participants found the two-interval task used in exp. 2 more challenging than the odd-one-out paradigm of exp. 1. Some evidence for this explanation comes from the observation that the error bars around the thresholds were typically larger in exp. 2 than in exp. 1. One reason why this may have occurred is that, in exp. 2, listeners had to identify the direction of the change, rather than detect any change at all. This would be consistent with the fact that some normal hearing (NH) listeners have difficulty in identifying the direction of the pitch change between two notes, even though they can tell that they have different pitches (Semal and Demany 2006). Even though we know of no similar finding for the perception of modulation depth (wobble), the requirement to focus on a particular cue (pitch or wobble) and to identify the direction of the change, whilst ignoring loudness differences, may have proved more challenging than simply detecting the odd one out using any cue, as in exp. 1.

## EXPERIMENT 3: MODULATION DEPTH DISCRIMINATION WITH EQUAL-RMS STANDARD AND SIGNAL

### Rationale

**Fig. 5:**
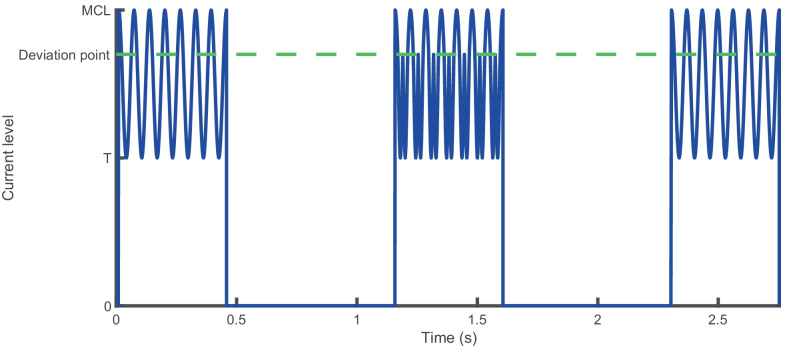
Stimulus modulation envelopes for experiment 3. In this trial, the signal stimulus is in the second interval

Experiment 3 used a novel method for measuring encoding of the stimulus envelope at levels below the peaks of the modulator. The method used an odd-one-out procedure as in experiment 1, thereby obviating the need to instruct the listener what cue to listen for, whilst precluding the use of loudness cues. The rationale is based on the observation that, although the loudness of AM electrical pulse trains depends on carrier rate and modulation depth, it does not depend on modulation frequency, for the range of modulation frequencies applied here (Chatterjee and Oberzut 2011; Fraser and McKay 2012), just as with acoustic hearing (Moore et al. 1999; Zhang and Zeng 1997). The likely reason why modulator rate does not affect loudness is that it does not affect either the RMS or the peak level of the stimulus; indeed, the distribution of instantaneous amplitudes is independent of modulator rate. Figure 5 illustrates a new set of stimuli for which these conditions are also met. Above a certain deviation point (dashed green line imposed on the signal envelope), the envelopes of the standard and signal stimuli are identical. Below the deviation point, the signal envelope is equal to the standard envelope compressed in time by a factor of 2, and repeated once. When the deviation point is equal to the modulator peak, the difference between the standard and signal envelopes corresponds simply to a doubling in modulation rate. Previous research has revealed that, at least at low-to-moderate modulation rates, this modulation-rate doubling is easily detected (Chatterjee and Oberzut 2011); this finding was confirmed here in an auxiliary experiment involving three participants. By adaptively varying the deviation point, experiment 3 measured the range of the modulator envelope over which subjects can reliably detect this envelope-rate doubling.

### Method

#### Participants

The same participants as in exp. 1 took part, except that participant M4 was unavailable and an additional subject, C7, was recruited in Cambridge.

#### Stimuli and Hardware

Standard stimuli were identical to those in exp. 1 except that in addition to the 1000-pps carrier rate, some participants also performed the experiment with a 6000-pps rate. A high pulse rate was required to test the highest modulation rates (> 62.5 Hz) for this experiment because the complex shape of the stimulus meant that the waveform would be under-sampled at the highest modulation rates with a 1000-pps carrier. For the 1000-pps carrier, the experiment used a restricted range of modulation rates from 15.625 to 62.5 Hz. For the 6000-pps carrier, a wider range of modulation rates from 7.125 to 250 Hz was tested. This was made possible by the use, for all conditions, of a CP910 processor and NIC4 software provided by Cochlear Ltd. This allows low-latency streaming for indefinite time, removing the buffer size limitations with NIC2. The stimulus duration for the 7.8125-Hz condition was 0.512 s, giving 4 cycles of modulation for the stimulus. The 7.8125-Hz condition was also tested at a stimulus duration of 1.024 s for some participants, in order to determine whether performance was limited by the small number of cycles presented at the 0.512-s duration.

Signal stimuli were constructed to be identical to the standard above a variable ‘deviation point’ (see Fig. 5). Below the deviation point, the stimulus trough was duplicated and compressed in the temporal dimension by a factor of 2 (effectively doubling the rate of this portion of the waveform). Note that when the deviation point was at MCL, corresponding to the peak of the envelope, the difference between the signal and standard was simply a doubling in modulation rate.

For 1000-pps stimuli, the same loudness estimation procedure as for exp. 1 and exp. 2 was used to determine thresholds and MCLs. For 6000-pps stimuli, thresholds and MCL were measured for an unmodulated 6000-pps stimulus, using the same loudness estimation procedure. Modulated 6000-pps stimuli were then based on these levels for each subject.

An auxiliary experiment measured performance as a function of the deviation point using the method of constant stimuli. The carrier rate was 1000 pps, and modulation rates of 15.625, 31.25, and 62.5 Hz were tested. Participants M1, M2, and M3 took part, using the same threshold and MCL levels as for the main experiment.

#### Procedure

As in exp. 1, a 3-interval 2-alternative forced-choice paradigm was employed. Participants were instructed to use any cue to make their judgement, and an adaptive procedure, similar to that employed in experiments 1 and 2, was used to estimate the threshold in the main part of the experiment. The auxiliary experiment also used the same 3-interval 2AFC design as the main experiment. For each subject and modulation rate, a range of modulation depths were selected, based on performance in the main experiments. A modulation depth corresponding to 0% (unmodulated) was always included (expected to give 100% performance), together with between two to five additional modulation depths. Testing took place in blocks of a particular modulation rate, with 20 presentations of each modulation depth, in random order. One or two blocks were measured for each modulation rate, with blocks presented in random order. Each modulation rate could thus be expressed as a psychometric function with scores out of 20 or 40 per modulation depth.

### Results

**Fig. 6:**
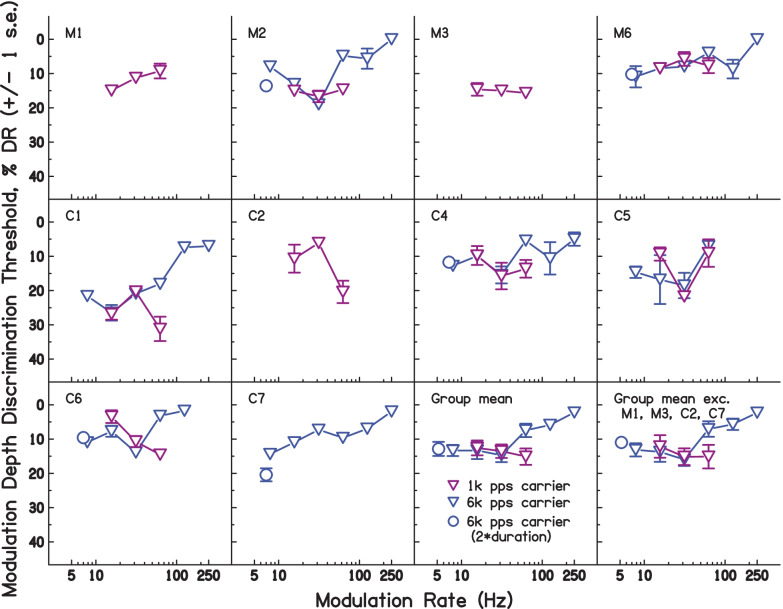
Experiment 3 results for the 1000-pps and 6000-pps carrier rate conditions. Individual subject data show ± 1 s.e. of the mean of trials making up each condition. Group mean data show ± 1 s.e. of the group mean. Bottom-right panel shows mean from subjects who completed both rates

Results are shown for the 1000-pps and 6000-pps conditions by the magenta and blue symbols, respectively, in Fig. 6. Strikingly, performance was extremely poor for both pulse rates; for modulation rates between 15.625 and 62.5 Hz, average thresholds were 14% and 12% for the 1000- and 6000-pps conditions, respectively. For the higher modulation frequencies in the 6000-pps conditions, average thresholds were just 5%. This means that subjects were unable to detect a doubling in the modulation frequency occurring in the bottom 90–95% of the envelope.

To evaluate the effect of modulation frequency, LMMs were fitted separately for each pulse rate with a modulation frequency as a continuous variable and a random intercept for each subject. For the 1000-pps rate (modulation frequencies: 15.625, 31.25, 62.5 Hz), the main effect of modulation frequency was not significant (*χ*^2^(1) = 3.27, *p* = 0.0708). For the 6000-pps rate, where the full range of modulation frequencies was tested, there was a significant main effect of modulation frequency (*χ*^2^(1) = 57.25, *p* < 0.001) with a negative regression coefficient (*β* =  − 0.0517). This indicated that performance with the 6000-pps carrier deteriorated with increases in modulation frequency, being generally better for modulation frequencies of 62.5 Hz and below. At the lowest modulation frequency, 7.125 Hz, doubling the stimulus generation to yield 8 rather than 4 cycles of modulation did not substantially affect performance, as shown by the unconnected circles to the left of each panel of Fig. 6.

**Fig. 7:**
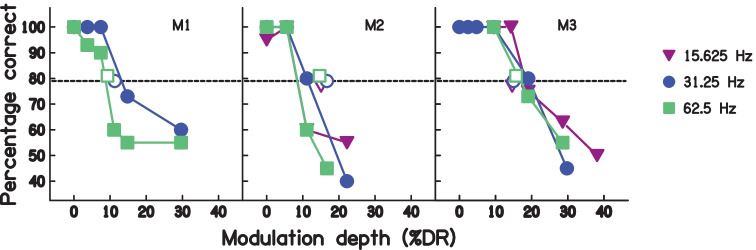
Psychometric functions relating performance to modulation depth for M1, M2, and M3 for rates 15.63, 31.25, and 62.5 Hz. Note different abscissa for each subject. Open symbols show result obtained from main (adaptive) task at each rate

Figure 7 shows the psychometric functions obtained in the auxiliary condition with a pulse rate of 1000 pps. When the deviation point corresponded to a modulation depth of 0%, the task was equivalent to detecting a doubling of modulation rate, and performance was perfect, or nearly so, for all three subjects and all three modulation rates tested. Performance dropped markedly as the modulation depth exceeded a certain amount, which depended somewhat on the participant and the modulation rate. It was generally better for participant M3, who could perform the task well for modulation depths up to 15–20%, than for the other two participants. The psychometric functions were monotonic for all participants and modulation frequencies, thereby validating the use of an adaptive procedure in the main experiment. The horizontal dashed line indicates the 79% correct point on the psychometric function; this is the point on which the adaptive procedure, used in the main experiment, theoretically converged. The results are broadly consistent with the adaptive procedure in that they predict thresholds of less than 20% modulation depth, and that the effect of modulation frequency is greater for M1 than for the other two subjects. Discrepancies include the fact that the predicted thresholds for M2 correspond to somewhat smaller modulation depths than obtained in the adaptive procedure of the main experiment (open symbols in Fig. 7).

## DISCUSSION

### Comparison with Previous Experiments

Two previous studies assessed modulation depth discrimination in CI listeners. Using an odd-one-out procedure, Busby et al. (1993) measured MDTs employing a standard consisting of an unmodulated train of pulses, each with phase duration of 100 µs, and a signal of the same current level but with phase duration amplitude modulated sinusoidally. For pulse rates of 1000 pps, performance was good (thresholds were low), corresponding to less than 10–20% of the participants’ dynamic range at all rates. Thresholds increased, however, with increasing modulation rate, consistent with the pattern typically observed in MDT tasks (Fraser and McKay 2012; Shannon 1992). A similar standard was employed in assessing modulation depth discrimination, but with phase duration sinusoidally modulated by 50% around a duration of 100 µs, and the signal modulated by a greater amount. Here, in contrast to the relatively poor (compared to MDT) performance we observed, Busby et al. (1993) reported the same (low) thresholds for their MDDT task. This inconsistency may arise from differences in the way modulation depth was manipulated. In contrast to Busby et al. (1993) — who increased modulation depth by decreasing the minimum phase duration and, importantly, increasing the maximum phase duration during the signal interval — we maintained a constant modulation peak in standard and signal intervals, exploring listeners’ sensitivity to the troughs of the envelope. This meant that Busby et al.’s listeners could perform the depth discrimination task by detecting a difference in charge near the peak of the envelope.

Gomersall et al. (2016) presented contiguous frequency bands of noise, each of which was amplitude modulated stochastically, both to NH listeners and via the auxiliary input of the MedEL device to CI listeners. The stochastic modulator consisted of a low-pass noise, whose cut-off could be varied to control the average modulation rate. An expansive function was applied to vary modulation depth. An ‘oddest-one-out’ procedure was used, where independent modulators were selected for all three intervals and with statistics (e.g. average modulation depth) differing in one interval compared to the other two. Modulation depth discrimination was worse when the cut-off frequency of the low-pass modulator was 5 Hz than when it was 34 Hz, reminiscent of the deterioration in MDDTs observed at the lowest modulator frequency (15.6 Hz) in our experiment 1.

Previous studies with cochlear implant users support our findings of relative insensitivity to the lower portion of the dynamic range. Relevant to the results we observed at the highest modulation rates, Vandali et al. (2013) measured the rate of unmodulated pulse trains judged to produce a pitch equal to that of 1800-pps pulse trains sinusoidally amplitude modulated at rates of 100, 200, or 300 Hz. At a low modulation depth (12.5%), pulse trains evoked a pitch percept higher than their modulation rate, whereas at modulation depths of 50% and 100%, the pitch was close to the modulation rate. Matched pitches were significantly lower for 50% and 100% modulation depths compared to 12.5%, but did not differ significantly from each other, consistent with listeners being sensitive to portions of the envelope between 12.5 and 50% down from the peak of the envelope, but not lower than this. Carlyon and Deeks (2015) compared the pitch of a 200-pps pulse train, in which even-numbered pulses were attenuated, to that of unmodulated pulse trains of various rates. The largest modulation depth (1.36 dB) generated matches close to 200 pps for three subjects, but resulted in a drop in pitch for two others. With a dynamic range for a 200-pps pulse train presented in monopolar mode, as in Carlyon and Deeks (2015), of about 5 dB, this suggests that those two subjects were sensitive to differences in the top 27% of the dynamic range. Hu et al. (2017) tested the sensitivity of CI users to interaural time differences conveyed by 200-pps pulse trains, modulated at a rate of 20 Hz, and of normal hearing listeners to ITD cues in 200-Hz tones, also modulated at 20 Hz. CI users were sensitive to ITDs when cues were present only at envelope peaks but not at all sensitive to ITD when cues were only present in the rising slopes of envelopes. In contrast, normal hearing listeners were marginally more sensitive to cues presented in rising envelope slopes compared to those conveyed at envelope peaks.

It is also the case for normal hearing listeners that sensitivity to changes in modulation depth is poorer for greater reference modulation depths compared to shallower ones (i.e. sensitivity to change is poorer at the bottom compared to the top of the dynamic range). However, the difference is considerably less dramatic than for CI users. Ewert and Dau (2004) showed that in normal hearing listeners, Weber’s law holds for modulation discrimination for modulation depths greater than − 15 dB, with discrimination threshold increasing proportionally with increasing modulation depth. For normal hearing listeners, the threshold modulation index for discriminating a less-modulated 16-Hz sinusoid from a fully modulated one was 0.91 (Fleischer 1980). For comparison with the current study, converting the percentage of the dynamic range to the modulation index, the best performance in experiment 1 — ~ 75% of the dynamic range — corresponds to a threshold modulation index of 0.6. The best performance in experiment 3 — ~ 25% of the dynamic range — corresponds to a modulation index of 0.14 (if the stimulus were a sinusoidal signal). This is in contrast to performance in modulation detection tasks in which thresholds are similar or sometimes superior for CI users compared to normal hearing listeners (Bacon and Viemeister 1985; Shannon 1992).

### Effects of Temporal Smoothing on Sensitivity to Modulator Shape and Depth

Our data indicate that cochlear implant users are largely insensitive to changes that occur in the lower-amplitude portion of stimulus envelopes. This was particularly striking in experiment 3, where participants were unable to detect a doubling of modulator rate applied to the bottom 80–90% of the envelope. Several features of our results are explicable in terms of temporal envelope smoothing, a concept used to account for a range of observations in NH and CI listeners (Oxenham 2001; Plack et al. 2002; McKay et al. 2013). Envelope smoothing and temporal integration can be modelled by passing simulated neural activity through a temporal window. The output of the temporal window builds up over time, which is intended to account for backward masking, and decays after the input has ceased, accounting for forward masking (Oxenham and Moore 1994). Although the temporal integration model was initially employed to model normal hearing, its application to temporal processing in cochlear implant users is motivated by the similar timescale of forward masking in cochlear implant and normal hearing listeners (Shannon 1990)—notwithstanding a greater variability between CI subjects (Chatterjee 1999)—and the assumed central origins of backward masking (Elliott 1971; Plack and Viemeister 1992; Puleo and Pastore 1980). For CI listeners, this temporal integration approach has been used to model the effect of inter-pulse intervals on detection thresholds and loudness, the temporal modulation transfer function, the effect of duration on detection thresholds, and the decay of forward masking (McKay et al. 2013).

**Fig. 8:**
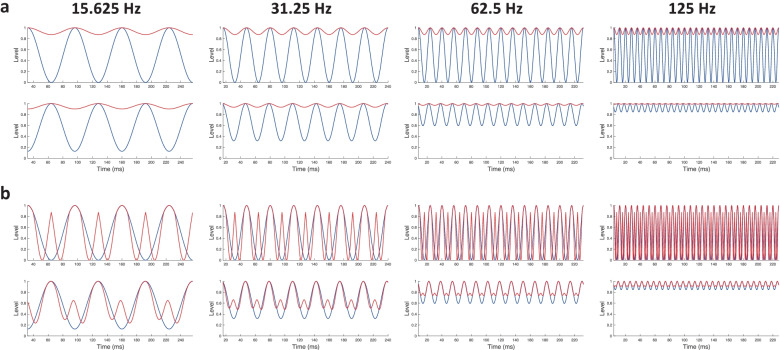
(**a**) Signal (red) and standard (blue) stimulus envelopes for experiments 1 and 2 for modulation rates from 15.625 to 125 Hz before (top) and after (bottom) the temporal window function has been applied. (**b**) Stimulus envelopes for experiment 3 before (top) and after (bottom) temporal smoothing

The top row of Fig. 8a shows the envelopes of the standard (blue) and signal (red) stimuli of experiments 1 and 2, for modulator rates ranging from 15.625 to 125 Hz, from left to right. The signal modulation depth is 12.5%, which would be easily detectable in both experiments (see bottom right-hand panel of Fig. 4). The second row shows the same envelopes after smoothing by a temporal window (McKay et al. 2013; Oxenham 2001) applied directly to the stimulus envelope, as in Lamping et al. (2020), rather than to simulated neural activity as in McKay et al. (2013). The window consists of two back-to-back exponentials and has an equivalent rectangular duration of 7.1 ms:

1 $$W\left(t\right)=\left\{\begin{aligned}\left(1-r\right){e}^{\left(\frac{t}{{T}_{b1}}\right)}+{re}^{\left(\frac{t}{{T}_{b2}}\right)}, t<0\\ {e}^{\left(\frac{t}{{T}_{a}}\right)}, t\ge 0\end{aligned}\right.$$

where *T*_*a*_ = 3.5 ms, *T*_*b*1_ = 4.6 ms, *T*_*b*2_ = 16.6 ms, and *r* = 0.17. It has almost no effect at the lowest modulation rate of 15.625 Hz, but at 32.5 Hz, the window smooths the troughs of the 100% modulated standard, and to a lesser extent of the 12.5% modulated signal. The smoothing effect is even greater at the highest two modulation rates. We refrain from attempting a quantitative model of the effect of modulator rate, because the nature of the task is clearly different for a 15.625-Hz modulator, where listeners can hear a change in the amount of wobble, than for a 125-Hz modulator, where listeners likely use a pitch cue. However, the temporal window clearly affects the envelope more at high than at low rates, and is therefore likely to contribute to the decrease (worsening) in thresholds as modulation rate is increased.

The effect of the temporal window is particularly informative in interpreting the results of experiment 3. The deviation point of 12.5% shown in Fig. 8b corresponds roughly to the average thresholds obtained for modulation rates up to about 62.5 Hz in experiment 3 (see Fig. 6), and so would have been only just detectable. Figure 8b also shows that, with a 12.5% deviation point, the signal and standard would not be discriminable at a modulation rate of 125 Hz. The top row of Fig. 8b shows that there is a secondary peak in the signal envelope (red), mid-way between the peaks of the standard (blue), which is greatly affected by the temporal window (see bottom row of Fig. 8b). The peak is attenuated at 15.625 Hz, reduced by an elevation of the envelope trough at 32.5 Hz, and largely eliminated at higher modulation rates, consistent with the very poor performance observed at the highest modulation rates in experiment 3, where, for the 125-Hz rate, participants were insensitive to changes occurring in the bottom 95% of the dynamic range. Importantly, at all modulation rates, the difference between the standard and signal envelopes is much smaller in Fig. 8b than in Fig. 8a, consistent with the lower (worse) thresholds in experiment 3. This illustrates the point that temporal smoothing, possibly related to forward and backward masking, can largely obscure seemingly obvious differences in the temporal envelope, whenever those differences occur in portions of the envelope that are below the peaks.

### Limitations

Two limitations of the study are worth noting. One is that the overall level of the thresholds differed substantially between the three experiments. For example, at a modulation rate of 31.25 Hz, where performance was best in experiment 1, thresholds were approximately 71, 50, and 15% in experiments 1, 2, and 3, respectively. This makes it hard to make a simple statement of the proportion of the envelope range accessible to the listener. Rather, it appears that the answer depends both on what aspect of the percept counts as ‘accessible’ and on the specifics of the stimulus change that is to be discriminated. For example, the discrepancy between the thresholds in experiments 1 and 2 could arise if the portion of the envelope between 55 and 75% down from the peak contributed to overall loudness, but did not affect the listener’s estimate of modulation depth. As shown in Fig. 8 and discussed above, the lower (worse) thresholds in experiment 3 compared to those in experiment 2 may be due to the more subtle change in the envelope introduced by the signal, which was more susceptible to smoothing by the temporal window.

### Practical Applications

The present results show that, for single-channel CI stimulation, CI listeners are insensitive to stimulus differences that occur over a substantial portion of the envelope, below that occurring at the peaks. Removing such undetectable pulses in CI speech-processing strategies may, in principle, be advantageous for two reasons. One of these is that current spreads between stimulating electrodes, and it is possible that pulses from multiple electrodes, although each undetectable alone, could combine to disrupt the responses of neurons located near other intra-cochlear electrodes. This could occur because many modern processing strategies use very short intervals between pulses on different channels, and because the currents applied to these pulses can interact at the level of the auditory nerve membrane (Boulet et al. 2016; de Balthasar et al. 2003; Guérit et al. 2020, 2018; Macherey et al. 2017; Middlebrooks 2004). A second possible advantage could arise from power savings, which could lead to extended battery life. However, removal of too many pulses may degrade the representation of the envelope in ways that CI listeners *can* hear. It is therefore useful to have basic information on the portions of the envelope that are and are not audible.

Two strategies that are relevant to the present study are fundamental asynchronous stimulus timing (FAST) (Smith et al. 2014) and temporal integrator processing strategy (TIPS) (Lamping et al. 2020). The FAST strategy identifies the peaks in the envelopes in each channel and replaces each peak with a single pulse. Preliminary evidence from five participants demonstrated that speech perception was not different compared to the subject’s clinical ACE settings, but that FAST could significantly improve the detection of ITDs for bilateral CI users. This is quite an extreme approach in that it removes all envelope information below the peaks. Our results show that, although listeners are insensitive to many changes in the troughs of the excitation pattern, changes in modulation were detectable within about 10–20% of the modulation peak for most listeners and CI rates, even in experiment 3, where thresholds were lowest (worst). A more nuanced approach was explored in the development of the TIPS strategy, which convolves the output of each channel of the continuous interleaved sampling (Wilson et al. 1991) strategy with the temporal window employed by McKay et al. (2013), and deletes any pulses whose removal would not change the window output by more than a criterion amount. Lamping et al. (2020) reported that speech perception in noise was improved when the criterion was such that 50% of pulses were removed, compared to the standard CIS strategy. More recently, Kludt et al. (2021) also used a temporal masking model to remove low-amplitude pulses and observed improvements in speech-in-noise perception relative to the MP3000 strategy.

## CONCLUSIONS

In contrast to the high degree of the sensitivity of CI users in detecting modulations (Shannon 1992), we have found that sensitivity to reductions in modulations relative to a fully modulated signal is very poor, particularly in the absence of any level cues. Experiment 3 additionally showed that when participants are required to make judgements based on changes to the portions of the stimulus following an envelope peak, only the very upper part of the dynamic range is sufficiently sensitive to make these judgements. The results of all three experiments are qualitatively consistent with the envelope in each channel being smoothed by the auditory system, using a temporal window similar to that used to model a wide range of phenomena in normal acoustic and cochlear implant stimulation. The effect of the window is to obscure the representation of the valleys of the temporal envelope. The results provide a first step towards a theoretical underpinning of methods to improve CI speech perception and/or reduce power consumption by removing inaudible but possibly deleterious pulses from the electrical stimulus.

## Data Availability

Data will be made available on request.

## Code Availability

Code for simulating temporal smoothing and generating stimuli will be made available on request.
